# The three-dimensional structure of wood enables horizontal water transport needed to conduct water around lesions

**DOI:** 10.1038/s41598-023-41817-8

**Published:** 2023-09-12

**Authors:** Peter Hietz, Sabine Rosner, Klaus Scheicher

**Affiliations:** 1 Institute of Botany, University of Natural Resources and Life Sciences, 1180 Vienna, Austria; 2 Institute of Mathematics, University of Natural Resources and Life Sciences, 1180 Vienna, Austria

**Keywords:** Plant physiology, Forest ecology

**arising from**: L. Dietrich et al.; *Scientific Reports* 10.1038/s41598-018-33465-0 (2018).

## Introduction

**Figure 1:**
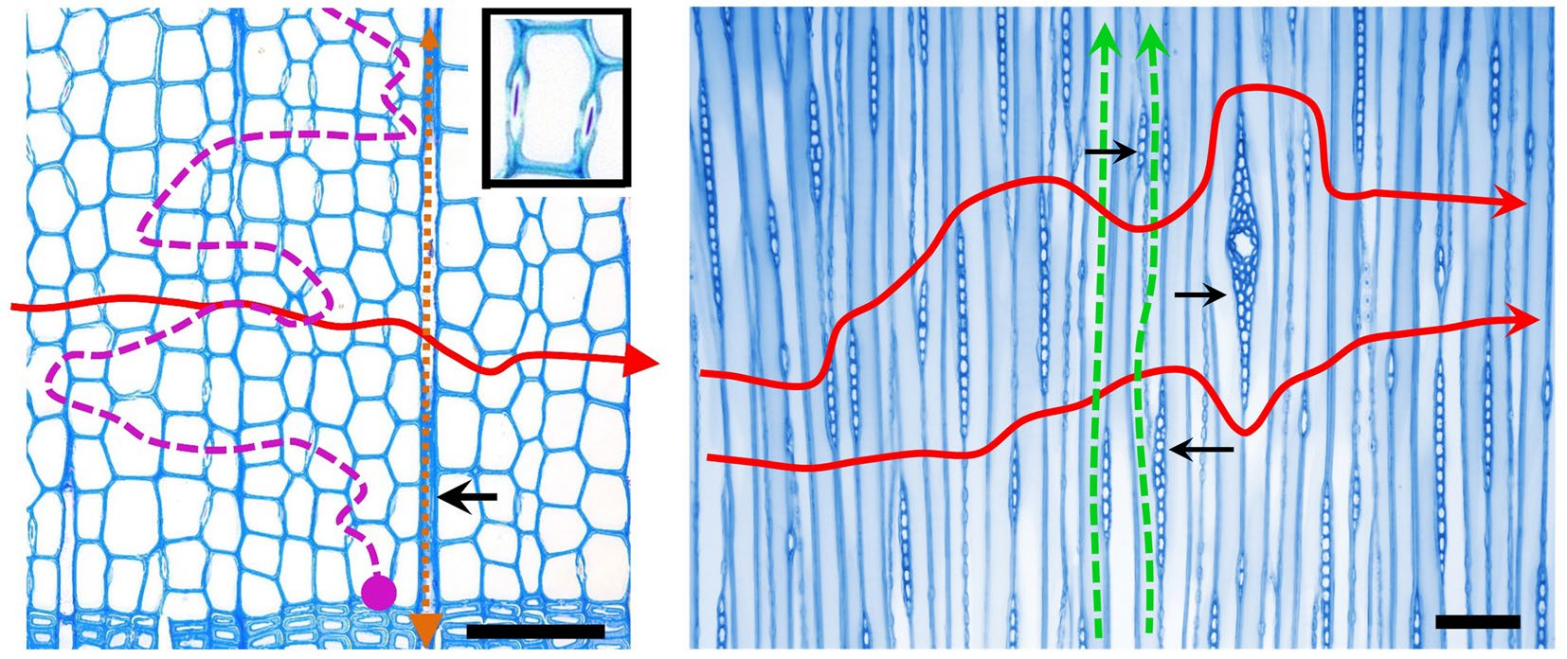
Possible flow paths in conifer wood. Images are from spruce (*Picea abies*) transverse (left) and tangential (right) sections (black scale bar is 100 µm). The insert shows an enlarged tracheid with typical bordered pits of conifers. Tangential flow (red solid line) can pass through pits in a rather straight line as all tracheids have pits to their neighbours on their radial cell walls (only some are visible in the section). These pits may also serve for radial flow (purple dashed line), but there are very few pits in tangential walls and few tracheids are connected to more than one tracheid on one side. The path would therefore be more tortuous and resistance is very high in latewood tracheids and at the ring boundary (purple circle^7^). Radial flow thus appears to pass mainly through the rays with high resistance (brown, dotted line). Right: rays (black arrows) are c. 100–400 µm high and may make the path of least resistance somewhat longer, but will not add very much resistance to radial flow (red arrow). Axial flow (green) needs to pass through pits much less frequently.

Land plants have evolved specialized conduits for water transport in their xylem, which is part of thin vascular bundles in herbs but forms compact wood in trees. These conduits are single-celled tracheids in the case of gymnosperms, typically 1–4 mm long and 20–80 µm wide, where the water needs to pass from one element to the next through small pores in the cell walls, the pits (Fig. 1), without which the resistance of the thick and lignified cell walls would be too high. The total resistance is substantial and composed of the resistance to flow within the conduits and the resistance through the pits between conduits^1^.

The transpiration of leaves supplied with water transported through a long stem creates a high water potential gradient along the transport pathway. This hydraulic system can be described in analogy to an electric current, where the flow rate is proportional to the potential gradient x conductance. If the water potential becomes too low as a result of high flow rates or low soil water potential, the liquid water under tension can suddenly vaporize (or cavitate), conduits become embolized, and water transport in the affected elements is blocked^2^. The resistance of the xylem to this embolism formation is thought to be an important aspect of plant drought resistance^3^ and the total conductance of the transport system must be sufficient to maintain water flow without the water potential becoming too negative^4^.

Dietrich et al.^5^ challenge this understanding. They reduced the conducting sapwood area in tree stems to half by cutting the main trunk with a chainsaw close to the base of the stem. Observing that this had little effect on stomatal conductance and water potential under high flow rates in summer they conclude that the transpiration of trees is not limited by their water transport capacity.

The expectation that cutting half the sapwood at one point along the path will reduce the total conductivity by 50% is correct if conduits are all parallel resistances without any lateral flow. However, while axial conductance will be reduced by half at the height of the cut, above and below the cut the entire sapwood area is available. Conduits are elongated to transport water in the axial direction, but as pits allow water to pass to neighboring elements, lateral flow must be possible (Fig. 1). How much the total stem conductance is affected when the conducting area is reduced to half at one point along the trunk, depends on the fraction of the total path length affected by the cut (which is very small) and how easily the water moves sideways to flow around the cut. If this is known, the effect of a wound, or more generally of non-uniform conductance within the stem, can be modelled as a network of elements with resistance varying with flow direction.

The axial conductance has been measured in many plants and is in the range^6^ of 1–500 kg m^−1^ MPa^−1^ s^−1^. Data on wood conductance in other directions are extremely scarce. Nevertheless, looking at xylem anatomy of conifers with elongated tracheids for axial transport and very few pits connecting tracheids radially suggests that radial conductance must be much lower than axial conductance and appears to be mainly conducted through rays^7^.

If the 3 cm-thick sapwood of a tree with 50 cm diameter is cut, as in the experiment by Dietrich et al.^5^, water needs to move to the wood above the cut mainly by tangential, and not by radial flow. We are not aware of any measurements of tangential conductance. However, for conifer wood this might be estimated from axial conductance and the anatomical structure. A typical spruce tracheid is 2 mm long and 30 µm wide in the tangential direction^8^ (Fig. 1). Tracheids overlap in their axial extension (but not tangentially) and are connected by bordered pits, most of which are on the radial cell walls. Therefore, water will be forced to move through one pit for approximately each 1 mm of axial flow, depending on how much they overlap^9^. In conifers, cell walls with pits contribute about 64% of total resistance in the axial direction with the rest accounted for by lumen resistivity^9^. For tangential flow, water passes through the same pits, but needs to pass through one pit every 30 µm, or 33 times across 1 mm. The resistance through the lumen will not be higher for tangential than for radial flow and only contribute a minor fraction of total tangential resistance. Thus, the resistance by the pits will be c. 33 times greater for tangential than for axial flow, and if pits account for 64% of axial resistance, tangential resistance would be c. 33 × 0.64 = 21 times greater than axial resistance. As parenchymatous ray cells probably have a higher resistance to tangential flow than tracheids and water will mostly flow around the rays (Fig. 1), this may add a bit to tangential resistance, but rays are not very high in conifer wood. Note that tracheids also overlap radially, thus some radial flow through the pits should also be possible (Fig. 1). However the resistance in the latewood is much higher^10^ and probably even higher at the ring boundaries where cells do not overlap radially. Conifers do also have pits on tangential walls, particularly at the ring boundary, and these may help to reduce the otherwise extremely high radial resistance^11^. In any case, our rough estimate for tangential conductance is probably not useful for radial conductance and we use literature data for the latter^12^. For our initial model we therefore assume that radial resistance is 2000 times greater and that tangential resistance is 20 times greater than axial resistance.

Dietrich et al.^5^ measured sap flow 10 cm centrally above the cut, directly opposite the cut, axially aligned 1.2 m above these two sensors and 5 cm sideways from the edge of the cut. These measurements provide a basis to test our model of hydraulic redistribution within tree stems.

## Results and discussion

**Figure 2:**
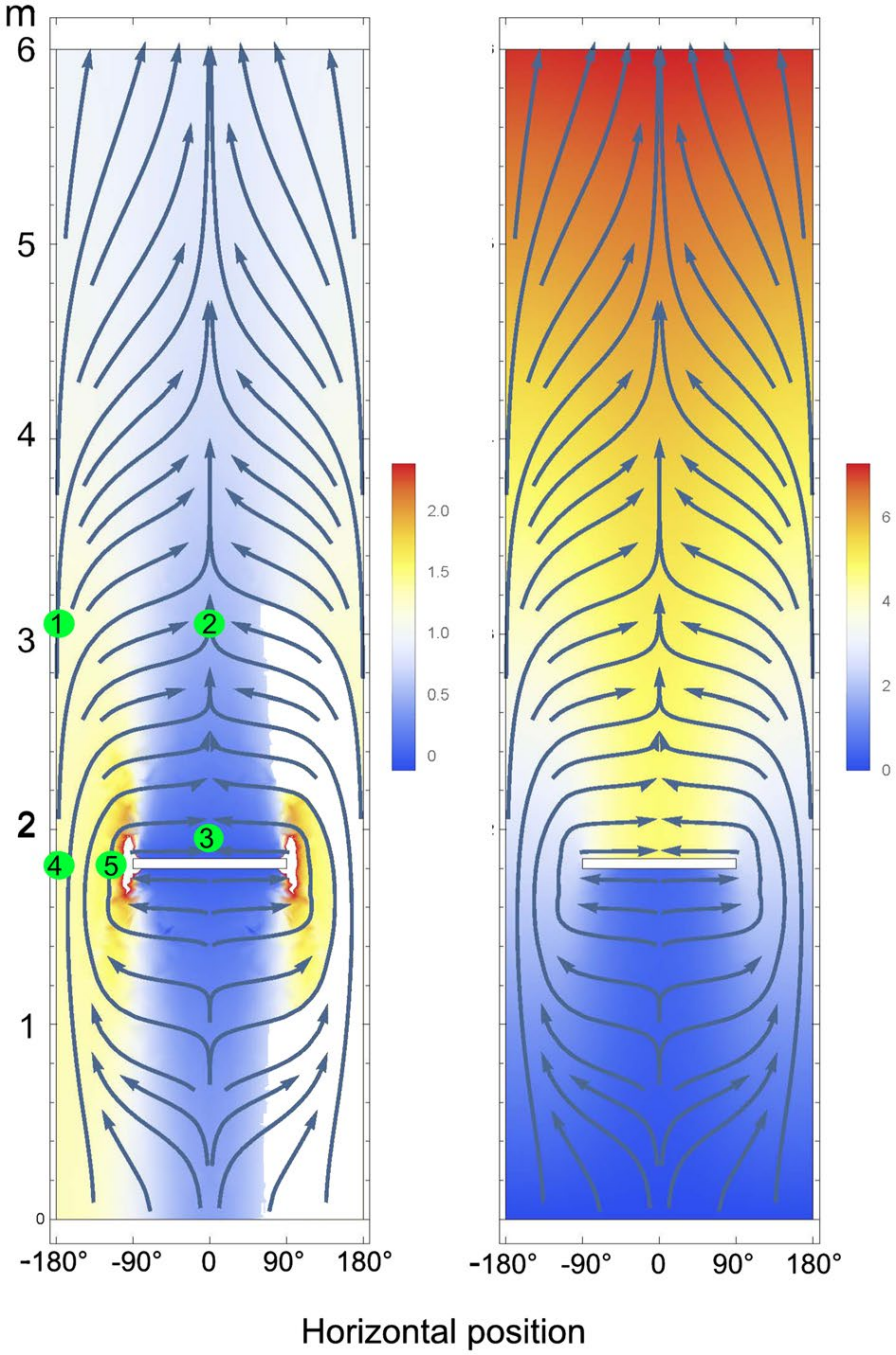
Model output of axial sap flow velocity (left) and water potential (right, both relative units) in a stem 1 m below to 4 m above a cut severing the sapwood in half of the stem (white notch). Arrows show the upward direction of water flow. Green dots in the left model represent points of sap flow measurements by^5^. Sap flow velocities > 3 (white) result from mathematical singularity at sharp 90° angles. Because radial conductance is very low and not relevant for the re-distribution of sap flow, the simplified model is two-dimensional with axial to tangential conductance 20:1 (see text for details).

At the point where the sapwood is cut, all water has to flow through the remaining half. The model shows substantial tangential flow in the stem section below and above the cut (Fig. 2) resulting in flux densities becoming more and more homogeneous over the length of the stem so that at 30 m virtually no effect was seen. While modelled flow opposite the cut increases by 36% relative to an intact stem, directly adjacent to the cut the flow rates increased by c. 77% (Supplementary Table S1A). This agrees with an increase in sap flow measured 5 cm next to the cut by c. 40% in spruce (*Picea abies*) and 200% in beech (*Fagus sylvatica*)^5^. Assuming axial conductance is 20 times higher than tangential conductance, sap flow 1.2 m above the cut was 48% of rates at the same height on the opposite side of the stem (Supplementary Table S1B). With axial conductance 10 or 30 times higher, modelled sap flow at this point was c. 63% and 40% of the sap flow at the opposite side, respectively.

The recovery of sap flow above a local lesion thus depends on the size of the interruption and the ratio of axial to tangential conductance. If the axial conductance is 20 times higher, losing half of the conductive area at 1.8 m results in a total reduction of sap flow of 7% and if axial conductance is 10 or 30 times higher, total flow would be reduced by 6% or 9%, respectively (Supplementary Table S1C). This also agrees well with the very small and non-significant changes in sap flow at a given water potential measured in spruce and beech. Cutting half of the sapwood would result in a 50% reduction of sap flow only if there were no horizontal re-distribution at all. Conversely, in our model even cutting 95% of sapwood at one point reduces the flow in a stem of 20 m length by only 39% (Supplementary Table S1D).

The experiment and measurements by Dietrich et al.^5^ thus provide an opportunity to test and refine our understanding of the hydraulic system of trees. Previous models of plant hydraulics^13^ have used variation in conductance along the axis, but not lateral variation. The latter is important to include for an anisotropic material such as wood, particularly in order to understand the effect of local interruptions. Starting from available data and assumptions based on wood anatomy, our model reproduces the measurement well and explains why Dietrich et al.^5^ failed to find a substantial reduction in water potential or conductance at the leaf level. It makes their interpretation of an over-capacity of the water transport system invalid and alternative explanations for the effect of hydraulic vulnerability or wood structure unnecessary. Measurements and model calculations of flux densities close to the cut both show a substantial local increase in flow rates. This requires higher water potential gradients, but only over a short distance, which hardly affects the water potential at the top of the stem. Thus, even a strong but local increase in flow does not require water potential to drop close to the cavitation threshold (compare the distribution of flow rates and water potential in Fig. 2). It has been argued^14^ that under severe drought the rhizosphere resistance is so high that xylem resistance is negligible under low sap flow demand. However, a model tested on different species that includes plant and soil conductance, rhizosphere resistance and xylem vulnerability showed that plants are xylem-limited but that the relative importance of rhizosphere versus xylem constraints depended on conditions such as soil texture and cavitation resistance^15^. In any case, neither our model nor their experiment addresses the total resistance along the soil–plant–atmosphere continuum as both explore the effect of changing the stem conductance.

The argumentation of Dietrich et al.^5^ more generally questions our understanding of water flow in plants being a function of the water potential gradient and conductance. Whether the water transport system has an over-capacity or not, if conductivity is reduced by half, flow rates must be reduced by half if the potential gradient remains constant or the potential gradient must double to maintain constant flow rates, which is counter to what was observed.

Our model of a stem is a rough approximation of the situation in a conifer tree, where the stem tapers, sap flux density varies radially^16^, branch junctions represent hydraulic constrictions^13^ and leaves can be decoupled from stem by high resistance to limit water loss from less redundant organs^17^. Spiral grain, in which conduits are aligned at a substantial angle to axial, also affects the re-distribution of sap flow, which may partially explain the functional significance of spiral grain^18^. Under most circumstances, tangential conductance appears to be much more important to re-distribute water and avoid catastrophic effects of wounds than radial conductance. This also explains why dye injection experiments^19^ commonly observe that the dye spreads faster tangentially within growth rings that radially.

In angiosperm wood, which is more complex than that of gymnosperms, the distribution of flow will also depend on the directional variation of conductance, but plausible assumptions are less straightforward than for our model representing conifer wood. In vessels, pits are distributed equally in the radial and tangential direction, but vessels mostly border fibers or parenchyma, and water flows mainly where two vessels, which form complex networks along the axis^20^, are in direct contact. While our model is based on the structure of conifer wood, the basic assumption that water can also be conducted horizontally is also true for angiosperms, even if the precise numbers are more difficult to come by. Without doubt a flexible water-transport system that allows for an adjustment of the flow path is important in all trees. Developing refined models reflecting the situation in angiosperms may also help to better understand the more complex structure and function in hardwoods.

## Model

Our initial model of a conducting stem is a hollow cylinder (hollow because the heartwood is not conducting) with a length of 30 m, a diameter of 50 cm and a sapwood thickness of 3 cm (Supplementary Fig. S1). We use a constant potential gradient of 1 from the base to the top and the relative differences in axial:radial:tangential resistances as 1:20:2000, and model water potential and flow rates in various directions along the stem. The absolute dimensions of the tree, flow rates and conductances are not relevant for the model as we were looking for relative changes in flux densities overall and at various points in the stem. Half of the diameter is cut at 1.8 m by removing one layer of elements over 5 cm.

Results of this model showed that radial conductance is irrelevant and a two-dimensional model produces the same results. We therefore choose a two-dimensional model on the surface of a cylinder, starting with axial conductivity 20 times greater than the tangential component. The corresponding partial differential equation, which is a weighted Laplace equation^21^, is

$$\frac{{\partial^{2} f}}{{\partial x^{2} }} + 400\frac{{\partial^{2} f}}{{\partial z^{2} }} = 0$$

where $\partial$ is the partial derivative, *f* is the water potential, *x* is the horizontal and *z* the axial distance, and 400 is the square of the axial to tangential conductivities.

With a radius r of 0.25 m, we parameterize the horizontal position as

$$0 \, \le {\text{ x }} \le \, 2{\text{r}}\pi \;{\text{m}}$$

And the axial position as

$$0 \, \le z \le \, 30\;{\text{m}}.$$

For an intact stem without a cut and homogeneous conductance the domain of this model, is the rectangle

$$R = \left[ {0,\;2r\pi } \right] \times \left[ {0,\;h} \right] = \left[ {0,\;1.57} \right] \times \left[ {0,\;30} \right].$$

We assume that in the intact stem the potential increases linearly with height, which leads to the Dirichlet boundary conditions^21^

1. $$(x,\;0) = 0$$ and
2. $$f(x,\;h) = h$$
  for 0 ≤ *x* ≤ 2rπ m. Due to the fact that there is no water lost, we assume the Neumann condition^21^
3. $$\vec{n} \cdot \nabla f(x,\;z) = 0$$
  on the rest of the boundary. The model output for an intact stem is a constant and homogeneous axial flow with a potential gradient of 1 from the base to the top (not shown).

For the model with a cut, we make the same assumptions on domain and boundary, but remove a rectangle from the interior of *R*. With the cut was set a height of *z* = 1.8 m with a length of *l* = 0.78 m (half the circumference) and a width of *Δ* = 5 cm, we obtain the rectangle

$$C = \left[ {\frac{1}{2}r\pi ,\;\frac{3}{2}r\pi } \right] \times \left[ {z,\;z + \Delta } \right] = \left[ {0.39,\;1.18} \right] \times \left[ {1.8,\;1.85} \right]$$

The domain for the model with cut is *R\C*. Along the boundary of the cut *C*, we again assume that there is no water exchange with the exterior and use the Neumann condition also on the boundary of the cut out rectangle.

Calculations were performed with the finite element solver of Wolfram Mathematica Version 11.3 (http://www.wolfram.com/mathematica, the source code is available from the authors). Mesh size, the density of points simulated, is selected automatically by Mathematica, but we increased mesh density near the cut and at the edge of the two-dimensional model because a fine-scale model resolution performs better for heterogeneous elements (Supplementary Fig. S1). We use the model to calculate flow rates in the x and z direction at positions corresponding to sap flow measurements by Dietrich et al.^5^, the water potential distribution and the effect of overall flow rates at a constant potential gradient. To assess the effect of variations in the ratio of axial to tangential conductance, the model was calculated with ratios of 10, 20 and 30. The total flow is the sum of all points through the cross-section. Since the model does not include capacitance, which does not pertain to the question, total flow is the same at any at any height.

### Supplementary Information

Supplementary Information.

## Data Availability

The Mathematica code is available from Klaus Scheicher (klaus.scheicher@boku.ac.at) on request.
