# Supplementary material for: The three-dimensional structure of wood enables horizontal water transport needed to conduct water around lesions
Source: Sci Rep. 2023 Sep 12;13:15057. doi: 10.1038/s41598-023-41817-8 (PMC10497525; doi:10.1038/s41598-023-41817-8)
Supplement: Supplementary file 1 — Supplementary Information. [file 41598_2023_41817_MOESM1_ESM.pdf]

Supplementary Information for

Matters arising

The three-dimensional structure of wood enables horizontal water transport needed to conduct water around lesions.

by Peter Hietz, Sabine Rosner and Klaus Scheicher

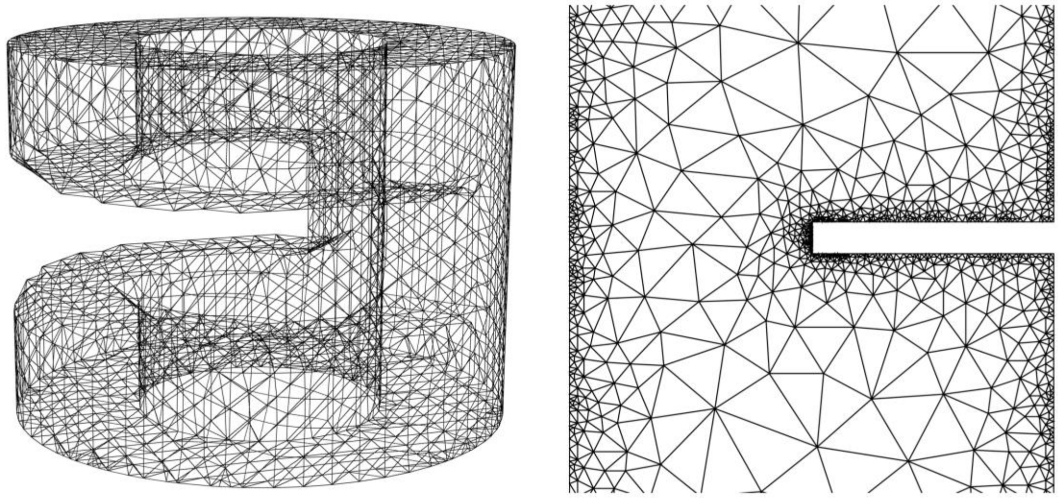

Supporting Figure 1 Left: Original 3-dimensional model a stem where the conducting sapwood is cut in half (variations in mesh density not shown). Right: adjusted mesh density at the cut and border for the 2-dimensional model. Only the stem close to the cut is shown here.

A

points in Fig.2	sap flow
1	1.19
2	0.57
3	0.06
4	1.36
5	1.77

B

axial:tang conductance	sap flow 1.2 m above cut	1.2 m opposite cut
10:1	0.71	1.13
20:1	0.57	1.18
30:1	0.48	1.21

C

axial:tang conductance	total sap flow
10:1	0.94
20:1	0.93
30:1	0.91

D

% sapwood cut	total sap flow
0	1
50	0.92
80	0.79
90	0.69
95	0.61

Supporting Table 1. Change in sap flow under various model conditions. All values are relative and scale linearly with model settings of total conductance or potential gradient. A: Sap flow at points indicated in Fig. 2. B: Sap flow in 3 m above a cut in 1.8 m, either opposite the cut (position 1 in Fig. 2) or directly above the cut (position 2). C: Sap flow relative to uncut tree depending on the axial relative to radial conductance in wood. D: Change in total sap flow when 0 – 95% of the sapwood was cut in 1.8 m.
